# Hermansky-Pudlak Syndrome: A Case Report

**DOI:** 10.1155/2014/249195

**Published:** 2014-02-20

**Authors:** Ilhami Berber, Mehmet Ali Erkurt, Irfan Kuku, Emin Kaya, Mustafa Koroglu, Ilknur Nizam, Mehmet Gul, Recep Bentli

**Affiliations:** ^1^Department of Haematology, Faculty of Medicine, Inonu University, 44280 Malatya, Turkey; ^2^Department of Histology and Embryology, Faculty of Medicine, Inonu University, 44280 Malatya, Turkey; ^3^Department of Internal Medicine, Faculty of Medicine, Inonu University, 44280 Malatya, Turkey

## Abstract

*Objective.* The aim of this paper is to report the case of a patient diagnosed with Hermansky-Pudlak syndrome, as a result of bleeding diathesis. *Clinical Presentation and Intervention.* A 23-year-old male presented with recurrent epistaxis and, upon physical examination, was found to be remarkable for albinism and suborbital ecchymosis. The absence of dense bodies in the platelets was demonstrated using electron microscopy. This patient was (slowly) administered one unit of a platelet suspension, and his bleeding decreased considerably. *Conclusion.* This case shows that Hermansky-Pudlak syndrome should be considered in the differential diagnosis of a patient presenting with bleeding diathesis, when the clinical presentation also includes oculocutaneous albinism and visual problems.

## 1. Introduction

Hermansky-Pudlak syndrome (HPS) is a hereditary multisystem disorder, characterized by oculocutaneous albinism and platelet storage deficiency, in which prolonged bleeding, pulmonary fibrosis, and granulomatous colitis may also occur. Although the first patients with this disorder were reported from Czechoslovakia, most of the subsequent patients have come from Puerto Rico [1]. There are nine types of human HPS reported to date, based on the genetic mutation from which the disorder stems [2]. Mutations observed in HPS are known to cause impairment of specialized secretory cells, including melanocytes, platelets, and lung alveolar type II epithelial cells. These patients demonstrate prolonged bleeding after surgical procedures and easy bruising [1]. The bleeding diathesis is variable; however, death from haemorrhaging has been reported [3]. Oculocutaneous albinism, the impairment of specialized secretory lysosomes in the melanocytes, is associated with HPS. The diagnosis is made by clinical findings of hypopigmentation of the skin and hair, eye characteristics, and the demonstration of absent dense bodies in the whole-mount electron microscopy of the platelets. The disease can cause dysfunction in the lungs, intestine, kidneys, or heart [1]. In this study, we report a case of HPS diagnosed as a result of bleeding diathesis.

## 2. Case Report 

**Figure 1:**
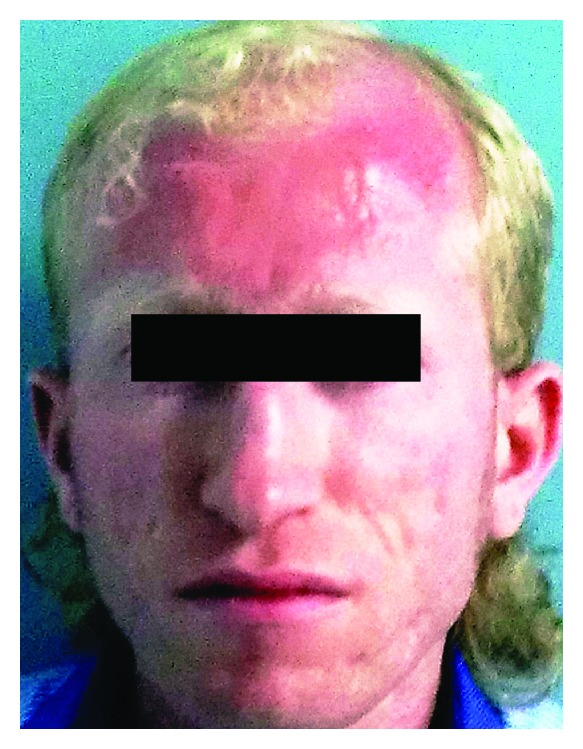
Patient exhibiting oculocutaneous albinism and exotropia.

**Figure 2:**
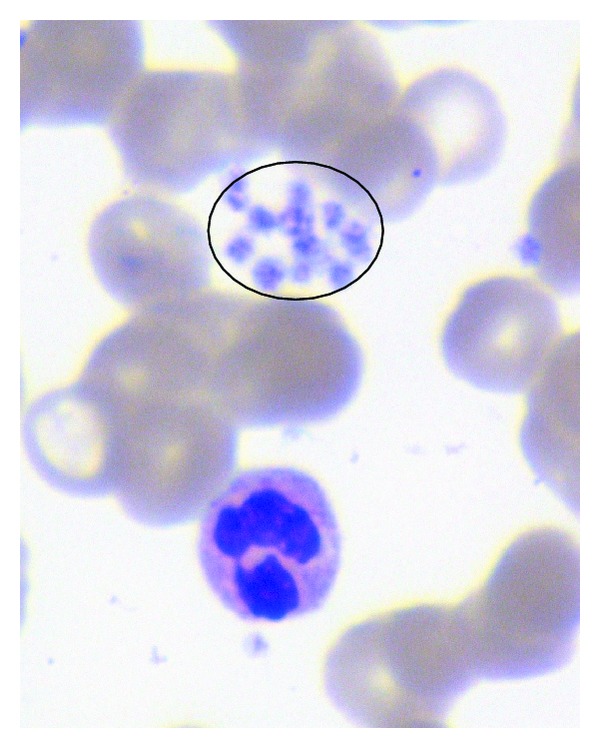
Abundant platelet clusters in peripheral smear.

A 23-year-old Turkish male with no significant disease history (with the exception of visual problems) presented with recurrent epistaxis after septoplasty operation. His past medical history indicated that he had bleeding episodes that were difficult to stop after circumcision and an appendectomy. He had no history of recurrent infections. His parents were unrelated, and he had one unaffected brother and sister. The physical examination was remarkable for albinism (definite skin and hair colour) (Figure 1) and suborbital ecchymosis. No additional petechiae, purpura, or ecchymosis was present on any part of his body. Upon ophthalmic examination he displayed nystagmus, exotropia, and foveal hypoplasia. Laboratory tests revealed a white blood cell count of 10 × 10^9^/L, haemoglobin level of 14 g/dL, and a platelet count of 252 × 10^9^/L. The fibrinogen, prothrombin time (PT), partial thromboplastin time (PTT), and platelet counts were normal; however, the bleeding time was prolonged. A peripheral smear examination revealed abundant platelet clusters and normal leukocyte distribution and red cell morphology (Figure 2).

**Figure 3:**
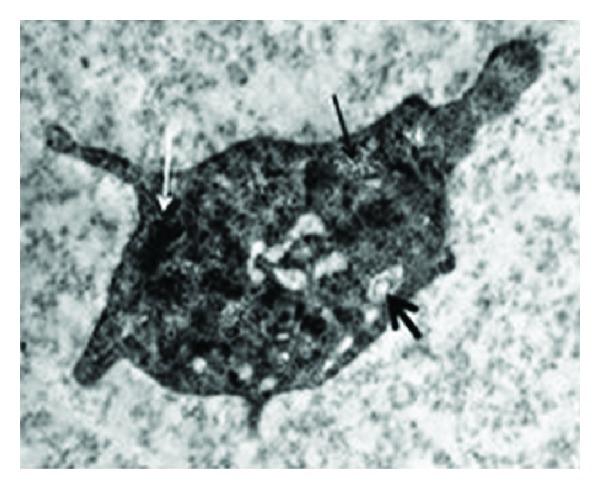
Dense bodies are absent in platelets with electron microscopy. Glycogen deposit area (thin arrow), open canalicular system (thick arrow), and electron-dense areas are not surrounded by membrane (white arrow), as observed in platelet cytoplasm.

A diagnosis of HPS was considered, based on the external and ocular features of albinism, visual disturbances, and bleeding diathesis. The classical method of platelet aggregometry testing was performed (Table 1). Upon electron microscopy, absent dense bodies on the whole-mount of the platelets were demonstrated (Figure 3). The von Willebrand factor activity was 76% (50–160%), the factor VIII activity was 96% (60%–150%), and the factor IX level was 129% (60%–150%). Due to technical difficulties, the test for genetic mutation on one of the genes known to cause HPS could not be performed. The patient was slowly administered one unit of a platelet suspension, and his bleeding decreased considerably. Due to ongoing bleeding, an additional unit of the platelet suspension was slowly infused, and his bleeding stopped.

## 3. Discussion

HPS, a rare hereditary disease caused by autosomal recessive inheritance, was first described by Hermansky and Pudlak in 1959. This disorder is found more commonly in individuals from Puerto Rico [1]. There are three main disorders caused by HPS, which result in the following symptoms: Albinism and eye problems. Albinism is a general term used for a heterogeneous group of diseases with genetic transmission, which occurs due to a disorder of the transformation of tyrosine to the melanin pigment. Individuals exhibiting albinism will have varying amounts of skin and hair pigment (melanin). The visual and ocular manifestations include photophobia, strabismus nystagmus, foveal hypoplasia, and impaired vision [1]. In our patient, a detailed visual examination added the diagnoses of myopia and strabismus, and glass eye prosthesis was recommended. Bleeding disorders: an examination of the thrombocytes revealed a marked decrease in the number of the dense granules in the platelets. These dense granules contain serotonin, adenine nucleotides, and calcium, which are necessary factors for normal platelet aggregation. It has been shown that the platelets of patients with HPS contain 10% less serotonin and ADP [4–6]. In our patient, although there were effective buffers anterior and posterior to the nose, bleeding could not be controlled during the twelve hours following a septoplasty operation. In the differential diagnosis of unexplained excessive bleeding, we considered inherited platelet function disorders, including Wiskott-Aldrich syndrome, Glanzmann's thrombasthenia, Bernard-Soulier syndrome, ADP receptor defect, von Willebrand disease, idiopathic dense-granule disorder, Hermansky-Pudlak syndrome, Chediak-Higashi syndrome, and grey platelet syndrome [7]. We settled on the diagnosis of HPS due to skin and hair hypopigmentation, the ocular findings, and bleeding diathesis. A classical method of platelet aggregometry was performed to diagnose HPS. In this method, a panel of platelet agonists (epinephrine, collagen, ADP, and ristocetin) at a range of concentrations triggers platelet activation and is used to detect storage-pool and secretory defects. The ristocetin aggregation and epinephrine secretion test results were abnormal; however, the von Willebrand factor activity, factor VIII level, and factor IX level were within normal ranges. We demonstrated absent dense bodies of the platelets on whole-mount electron microscopy. In order to stop the patient's bleeding, we considered platelet suspension apheresis, which is important in the treatment of patients with congenital platelet disorders. Platelet suspensions should be used selectively and sparingly because of the risk of alloimmunization against HLA antigens and/or platelet glycoproteins. HLA-matched single donors of platelets should be used to reduce this risk. If such donors are unavailable, leukocyte-depleted blood components should be used [8]. In this case, we were not able to detect HLA-matched donor platelets, so we used leukocyte-depleted blood components. The patient was slowly infused a total of two units of a platelet suspension, and his bleeding stopped with no complications. Cellular storage disorders: the syndrome causes a wax-like substance (ceroid lipofuscin) to accumulate in the body tissues and cause damage, especially in the lungs and kidneys, particularly in certain types of HPS [9].

There are nine types of HPS described to date, with mutations in HPS1-9 [2]. The HPS1 subtype is the most common HPS genetic mutation. Patients with HPS1 characteristically develop severe pulmonary fibrosis. Colitis occurs in about 15% of these cases. The HPS2 subtype has mild immunodeficiency symptoms with HPS morphological features. Patients with HPS3 mutations typically present with mild phenotypical features. The HPS5 subtype was found in one 3-year-old Turkish boy with HPS features, and HPS8 is characterized by reduced pigmentation [1]. We could not evaluate our patient for a genetic mutation due to technical deficiencies. Bleeding episodes of HPS patients may be severe enough to cause fatalities [3]. In order to prevent severe bleeding, desmopressin, plasmapheresis, or platelet suspensions may be used [4]. While there is no cure for HPS, treatment for the chronic haemorrhages associated with this disorder includes therapies with vitamin E and the antidiuretic desmopressin acetate [10].

This disease can also cause organ dysfunctions and multiorgan damage (lungs, intestine, spleen, liver, or heart) [1]. A preoperative chest disease consultation is required in HPS patients because of the possible postoperative pulmonary complications associated with this syndrome. Intraoperative, perioperative, and postoperative haemorrhage should be prevented and treated in patients with HPS. Desmopressin acetate and plasmapheresis can be used in the perioperative period [10]. HPS patients are advised to consult with pulmonary and haematology specialists in the preoperative period for potential lung or bleeding problems, respectively, during surgery.

## 4. Conclusion 

HPS should be considered in the differential diagnosis in patients presenting with bleeding diathesis, when the clinical picture also includes oculocutaneous albinism and visual problems.

## Figures and Tables

**Table 1:** Platelet aggregometry.

	% aggregation	Normal range
Platelet aggregation ristocetin (final concentration of 0.5–1.0^8^)	9%	>60%
Platelet aggregation collagen (final concentration of 2–5 µM)	65%	>60%
Platelet aggregation ADP (final concentration of 5–10 µM)	108%	>60%
Platelet aggregation epinephrine (final concentration of 0.5–10 µM)	44%	>60%

## References

[1] Pierson DM, Ionescu D, Qing G (2006). Pulmonary fibrosis in Hermansky—Pudlak syndrome: a case report and review. *Respiration*.

[2] Cullinane AR, Curry JA, Carmona-Rivera C (2011). A BLOC-1 mutation screen reveals that PLDN is mutated in Hermansky—Pudlak syndrome type 9. *American Journal of Human Genetics*.

[3] Davies BH, Tuddenham EG (1976). Familial pulmonary fibrosis associated with oculocutaneous albinism and platelet function defect: a new syndrome. *Quarterly Journal of Medicine*.

[4] Harrison C, Khair K, Baxter B, Russell-Eggitt I, Hann I, Liesner R (2002). Hermansky—Pudlak syndrome: infrequent bleeding and first report of Turkish and Pakistani kindreds. *Archives of Disease in Childhood*.

[5] White JG (2003). Electron-dense chains and clusters in platelets from patients with storage pool-deficiency disorders. *Journal of Thrombosis and Haemostasis*.

[6] Maurer-Spurej E, Dyker K, Gahl W, Devine DV (2002). A novel immunocytochemical assay for the detection of serotonin in platelets. *British Journal of Haematology*.

[7] Bolton-Maggs PH, Chalmers EA, Collins PW (2006). A review of inherited platelet disorders with guidelines for their management on behalf of the UKHCDO. *British Journal of Haematology*.

[8] Seligsohn U (2012). Treatment of inherited platelet disorders. *Haemophilia*.

[9] Dhaunsi GS (2005). Molecular mechanisms of organelle biogenesis and related metabolic diseases. *Medical Principles and Practice*.

[10] Wijermans PW, van Dorp DB (1989). Hermansky—Pudlak syndrome: correction of bleeding time by 1-desamino-8D-arginine vasopressin. *American Journal of Hematology*.

